# An Assessment of the Interindividual Variability of Internal Dosimetry during Multi-Route Exposure to Drinking Water Contaminants

**DOI:** 10.3390/ijerph7114002

**Published:** 2010-11-17

**Authors:** Mathieu Valcke, Kannan Krishnan

**Affiliations:** 1 Département de santé environnementale et santé au travail, Université de Montréal, CP 6128, Succursale Centre-Ville, Montréal, Québec, H3C 3J7, Canada; E-Mail: mathieu.valcke@inspq.qc.ca; 2 Institut national de santé publique du Québec, 190 Boulevard Crémazie Est, Montréal, Québec, H2P 1E2, Canada

**Keywords:** drinking water contaminants, inter-individual variability, liter-equivalents, multi-route exposure, physiologically-based pharmacokinetic modeling

## Abstract

The objective of this study was to evaluate inter-individual variability in absorbed and internal doses after multi-route exposure to drinking water contaminants (DWC) in addition to the corresponding variability in equivalent volumes of ingested water, expressed as liter-equivalents (LEQ). A multi-route PBPK model described previously was used for computing the internal dose metrics in adults, neonates, children, the elderly and pregnant women following a multi-route exposure scenario to chloroform and to tri- and tetra-chloroethylene (TCE and PERC). This scenario included water ingestion as well as inhalation and dermal contact during a 30-min bathroom exposure. Monte Carlo simulations were performed and distributions of internal dose metrics were obtained. The ratio of each of the dose metrics for inhalation, dermal and multi-route exposures to the corresponding dose metrics for the ingestion of drinking water alone allowed computation of LEQ values. Mean BW-adjusted LEQ values based on absorbed doses were greater in neonates regardless of the contaminant considered (0.129–0.134 L/kg BW), but higher absolute LEQ values were obtained in average adults (3.6–4.1 L), elderly (3.7–4.2 L) and PW (4.1–5.6 L). LEQ values based on the parent compound’s AUC were much greater than based on the absorbed dose, while the opposite was true based on metabolite-based dose metrics for chloroform and TCE, but not PERC. The consideration of the 95th percentile values of BW-adjusted LEQ did not significantly change the results suggesting a generally low intra-subpopulation variability during multi-route exposure. Overall, this study pointed out the dependency of the LEQ on the dose metrics, with consideration of both the subpopulation and DWC.

## 1. Introduction

When establishing drinking water guidelines (DWG, in mg/L) of chemical contaminants on the basis of non-carcinogenic effects, regulatory agencies account for the average daily ingestion rate of water (IRW, L/day) by an adult [1]:

(1) $$\text{DWG}=\frac{\text{TRV}\times \text{BW}\times \text{RCS}}{\text{IRW}}$$

where TRV is the toxicological reference value (in mg/kg BW_day), BW is the body weight of an average adult (*i.e.*, 70 kg), and RCS is the relative source contribution (20% by default) of drinking water to total exposure. For several drinking water contaminants (DWC) that are volatile and/or lipophilic, exposure can also result from the inhalation of vapors emitted from water as well as from the dermal contact during water usage for hygiene and domestic purposes. Such “multi-route exposure” has been well documented for trichloroethylene (TCE) and chloroform based on experimental data as the absorbed dose resulting from the inhalation of vapors and the dermal exposure to water could actually equal or even exceed the absorbed dose resulting from the ingestion of drinking water [2–7]. These results were also confirmed by dose estimates [8] and physiologically-based pharmacokinetic (PBPK) modeling [9]. Deterministic PBPK modeling has also been used to evaluate the contribution of inhalation and dermal exposure to internal doses and/or cancer risk of chloroform [10], tetrachloroethylene (PERC, [11]) and methyl-t-butyl-ether (MTBE, [12]).

The notion of “liter equivalent” (LEQ) has been defined as the amount of water that needs to be ingested to absorb an equivalent dose of DWC by other routes of exposure (*i.e.*, inhalation, dermal). As an example, an adult’s inhalation exposure to chloroform during an eight-minute shower has been estimated to generate an absorbed dose that corresponds to 17% of the total dose absorbed, which is equivalent to ingesting 0.46 L of water [8]. In addition, a ten-minute shower generated a LEQ of 0.50–0.57 L [6,9]. Dermal exposure has been estimated to contribute to 19 and 31% of the total absorbed dose in adults during a 10- and 30-min shower, respectively [9]. As a result, Haddad *et al.* [9] determined that multi-route exposure to chloroform, including ingestion, inhalation and dermal contact for 10- and 30-min showers resulted in absorbed doses equivalent to the doses resulting from ingestion of 2.65 and 4.65 L in a 70 kg adult. For TCE, values of 2.51 and 4.54 LEQ were respectively reported for 10- and 30-min showers.

LEQ values of 7.2 and 4 have been used by CalEPA [13] and Health Canada [14], respectively, in the establishment of drinking water guidelines for TCE. Also, a value of 4.11 LEQ was determined for trihalomethanes on the basis of data for chloroform [15]. For PERC, Health Canada has determined that the contribution of the dermal route is equivalent to that of the oral ingestion [16]. Such values were based on estimates of the absorbed doses in average adults. Haddad *et al.* [9] showed, however, that the LEQ values differ significantly when based on internal dose metrics (DM) such as the area under the blood concentration *versus* time curve (AUC) or the amount metabolized (Amet), instead of the absorbed dose. This issue appears important when considering the mode of action of a chemical underlying the toxicological reference values or health guidelines [17].

In addition, multi-route exposure and corresponding calculations of LEQ in subpopulations other than average adults have barely been examined. In this regard, Krishnan and Carrier [18] evaluated the contribution of inhalation and dermal exposure in representative children aged 6, 10 and 14 years, as well as representative adults, by a deterministic PBPK modeling approach. For a 30-min bath, the LEQ values for the sum of inhalation and dermal exposure in these subgroups were 1.45, 1.79, 2.14, and 2.61 LEQ, respectively, for chloroform. For TCE, LEQ values were also determined for a 10-year old child (1.9 LEQ), a 14-year old adolescent (2.25 LEQ) and a 70 kg adult (2.41 LEQ). Other subpopulations like pregnant women (PW) or the elderly have not been subjected to such evaluation, even though it appears logical that in the particular case of PW, their high inhalation rates and dermal surface area [19,20] could result in greater BW-adjusted intake of DWC via inhalation or dermal contact.

Therefore, since the inter-individual variability in absorbed and internal doses during multi-route exposure to DWCs has not been evaluated to-date, the objectives of this study were: to evaluate the inter-individual variability in absorbed and internal doses during multi-route exposure to DWC; and to estimate the corresponding variability in LEQ values.

## 2. Methods

The overall method involved using a physiologically-based pharmacokinetic (PBPK) model to compute distributions of LEQ values on the basis of probabilistic distributions of internal DM following a multi-route exposure scenario to known DWCs, namely chloroform and tri- and tetra-chloroethylene (TCE and PERC). Such distributions were obtained in various subpopulations (adults, neonates, children, elderly and pregnant women) by means of Monte Carlo (MC) simulations that account for the variability in the input parameters of the PBPK model.

### 2.1. PBPK Model Structure and Parameters for Specific Subpopulations

The previously published and validated PBPK model [21] for chloroform, TCE and PERC written in Microsoft Excel^®^(Microsoft Corporation, Seattle, WA) as per Haddad *et al.* [22] was used for this study. Briefly, this model consists of six basic compartments (liver, fat, skin, kidneys, richly perfused tissues and rest of the body), with a seventh compartment (foeto-placental unit) being added for pregnant women. As a useful feature with regard to the study of interindividual variability in internal dose metrics, the model ensures that physiological parameters are correlated for a given body weight/height, age and gender, while authorizing age-specific variations in the relationships between the physiological parameters and their determinants. To do so, this model framework includes mathematical equations that allow the calculation of physiological parameters as a function of four determinants, namely body weight, height, age, and gender. Additionally, a “variability term” based on data of the literature [23–26] is included as a multiplier of the results of selected physiological parameter values calculated with these equations in order to account for the variability in the physiology of two persons of identical age, gender and body mass index. Finally, first order metabolism is considered to occur mainly in the liver, with a minor contribution of kidneys in the case of chloroform and PERC. In computing the metabolism, the model allows the use of catalytic turnover of CYP2E1 (in pmol/mg of microsomal protein, MSP), the isozyme involved in the metabolism of the contaminants investigated and for which data on inter-individual variability are available [27,28].

Table 1 shows the statistics, explained in detail in previous work [21], describing the physiological determinants of the investigated subpopulations. These data were based on the P^3^M database (The Lifeline Group Inc, Annandale, VA) [29], as well as the literature [20,23–28,30–34]. Apart from adults (18–64 years), four presumably sensitive subpopulations were chosen for this study: neonates (birth–30 day), children (1–3 years), elderly (65–90 years) and pregnant women (PW; 38th week of gestation).

### 2.2. Exposure Scenarios and Dose Metrics Computed

For each subpopulation, a multi-route exposure scenario to each drinking water contaminant was simulated. Chemical-specific data taken from the literature are indicated in Table 2.

The multi-route scenario considered includes daily ingestion of drinking water (separated into five episodes spaced by a timelag of three hours) as well as inhalation of vapors in the bathroom and dermal contact with water during a once-daily 30 min bathroom exposure, as per the risk assessment of Health Canada [14,15]. The water-to-air transfer model used in the present study [47], described previously by Haddad *et al.* [9], does not differentiate the bathroom exposure to chemicals during shower from the bath. As the dermal contact and inhalation exposures are described based on the total volume of water used, the modeling results described here can be applied to both shower and bath. To calculate the air concentration of DWC in the shower stall, a water flow rate of 8.7 L/min [11] (rather than 10 L/min [47]), was used as this modification resulted in predicted air concentrations that were somewhat closer to the measured data of Jo *et al.* [3] (not shown).

The dermal surface to be in contact with water during the shower was considered to be 75% [48]. A 15 μg/L water concentration was retained since it would yield a daily dose of approximately 1 μg/kg/day for a 70 kg adult as per the risk assessment of Health Canada [14,15]. The computed DM included the total absorbed dose (Dabs), the 24-h area under the arterial blood concentration *versus* time curve for either the parent compound (AUC_pc_) or stable metabolite TCA (AUC_met_, for TCE and PERC), and the amount metabolized per 24 h per L of liver (Amet).

### 2.3. Calculation of LEQ Values

The LEQ values were calculated based on each DM. Thus, the LEQ value based on Dabs was calculated as:

(2) $$\text{LEQ}=\frac{\text{Dabs}}{\left[\text{DW}\right]}$$

where Dabs is the daily absorbed dose in μg/kg BW, and [DW] is the concentration in drinking water (*i.e.*, 15 μg/L), and LEQ is expressed in L/kg. For the calculation of the LEQ on the basis of internal DM (*i.e.*, AUC_pc_, AUC_met_ or Amet), the ratio of the internal DM for the multi-route exposure scenario (IDM_M-R_) and the internal DM for the ingestion exposure only (IDM_Ing_) was multiplied by the subpopulation-specific body-weight adjusted drinking water ingestion rate (IngR, in L/kg BW). It resulted in subpopulation- and DM-specific LEQ values, expressed in BW-adjusted values (L/kg BW):

(3) $${\text{LEQ}}_{\text{IDM}}=\frac{{\text{IDM}}_{\text{M}-\text{R}}}{{\text{IDM}}_{\text{Ing}}}\times \text{IngR}$$

The BW-adjusted LEQ value was then used to calculate the absolute LEQ value (in L) for individuals of a given BW in each subpopulation.

### 2.4. Probabilistic Modeling of Internal Dose Metrics for Multi-Route Exposure

For each subpopulation, MC simulations were performed using Crystal Ball® software (Oracle™, Redwood Shores, CA) in order to generate statistical distributions of internal DM after 2000 iterations. To avoid unrealistic combinations, BW and BH were correlated to 60% based on population distribution of body mass index in Canada [49]. The resulting DM distributions allowed the evaluation of the variability in internal DM (and corresponding LEQ) for the multi-route exposure scenario. As a measure of this variability, a “variability index” was computed as the ratio between the 95th percentile and (1) the median of DM in each subpopulation (“VI_spop_”) and (2) the median in adults (“VI”), for each contaminant investigated. This approach is similar to the approach of WHO on human kinetic adjustment factor (HKAF) [17,50].

### 2.5. Sensitivity Analyses

Sensitivity analyses on the model’s input parameters were performed to evaluate to what extent the different assumptions made with regard to these parameters affect the outcome of the model. Based on the impact on AUC_pc_, the sensitivity index (SI) for a given parameter (P) was calculated as:

(4) $$SI=\frac{{AUC}_{PC\_10}-AUC_{PC\_i}}{P_{10}-P_{i}}\times \frac{P_{i}}{{AUC}_{PC\_i}}$$

where subscript 10 denotes the AUC_pc_ and parameter (P) value when the latter is reduced by 10% compared to the initial value, indicated by subscript i. The greater the resulting SI value, the more influential is the parameter on AUC_pc_, while positive and negative SI values are linked to an increase and a decrease in AUC_pc_, respectively, when the parameter is increased.

## 3. Results

### 3.1. Simulation of Internal Dosimetry for Multi-Route Exposure

**Figure 1:**
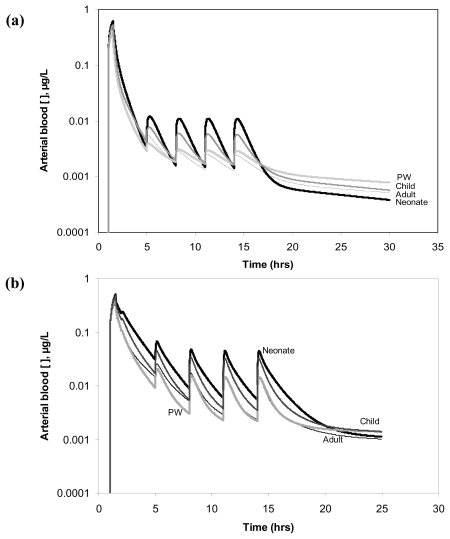

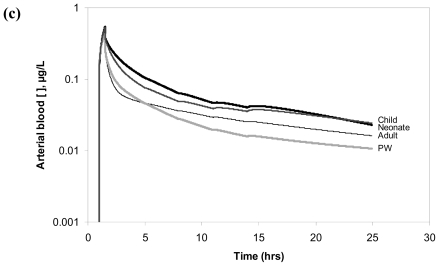
Model simulations of the arterial blood concentration of chloroform **(a)**, TCE **(b)** and PERC **(c)** during multi-route exposure in an average adult, neonate, child and pregnant woman (as per Table 1). The elderly are omitted since their profile is almost indistinguishable from an average adult’s. The scenario involves a 30 min bathroom exposure at t = 1 hr along with five episodes of drinking water ingestion at t = 2, 5, 8, 11 and 14 h.

The simulations of multi-route exposure shown in Figure 1 indicate that the neonates would have the highest blood concentration of parent compound as well as the fastest decline post-exposure.

**Figure 2:**
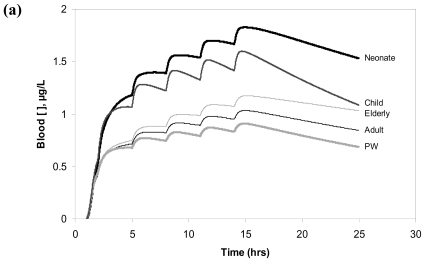

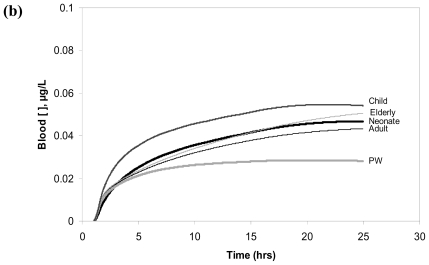
Model simulations of the blood concentration of TCA as a metabolite of TCE **(a)** and PERC **(b)** during multi-route exposure in average adult, neonate, child, elderly and pregnant woman (as per Table 1). The scenario involves a 30 min bathroom exposure along with five episodes of drinking water ingestion at t = 2, 5, 8, 11 and 14 h.

Children would have the second highest blood concentration of the parent compound, while adults and PW would have comparable profiles for chloroform and TCE. Blood concentration of PERC in PW appears constantly lower than that of adults. The contribution of the exposure resulting from the shower is always significant but more so for PERC as the ingestion episodes do not contribute significantly to increased blood levels likely because of its low K_o_ value. Conversely, absorption of chloroform and TCE during ingestion is fast, especially for the latter. With regard to TCA (Figure 2), blood concentrations resulting from the exposure to TCE (a) and PERC (b) are clearly greater in respectively the neonate and child than in the other individuals.

The model results also indicate that elderly would have higher blood levels of TCA than adults and even higher than PW. The contribution of TCE exposure via the shower to blood levels of TCA appears similar to the contribution of the ingestion events and is proportionally more important in the case of TCA produced by the metabolism of PERC.

**Figure 3:**
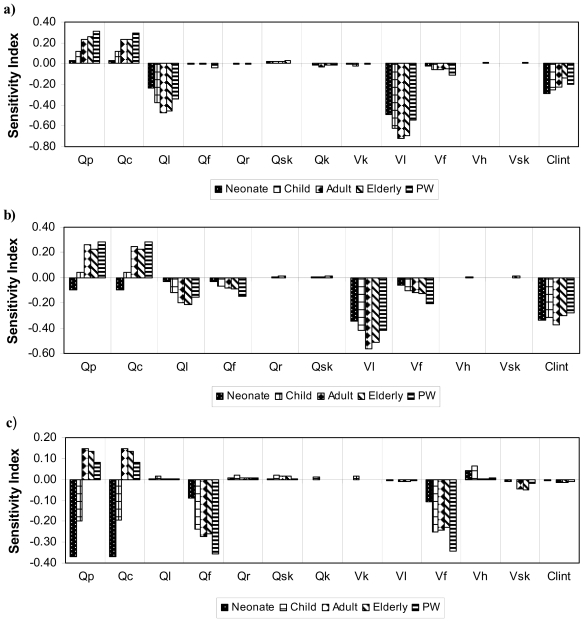
Sensitivity of parent compound’s area under the arterial blood concentration *versus* time (AUC, μg/L × hs) to the physiological parameters of the PBPK models for chloroform **(a)**, TCE **(b)** and PERC **(c)** in each subpopulation evaluated for a multi-route exposure to water contaminated with 15 μg/L. The sensitivity ratios were calculated as the change in AUC_pc_ for a 10% decrease in the value of input parameters (Clint, intrinsic clearance; Qp, alveolar ventilation rate; Qc, cardiac output; Qx, tissue blood flow and Vx tissue volume (l, liver; f, fat; r, rest of the body; sk, skin; k, kidney; h, highly perfused)), for a given age, body weight, body height and hepatic content of CYP2E1. In pregnant women, the volume of highly perfused tissues includes the feto-placental unit.

The results of sensitivity analyses are presented in Figure 3. They clearly show that considering every subpopulation and chemical under study, the most influential parameters of the PBPK models on the AUC_pc_ following a multi-route exposure are liver volume and blood flow (for highly metabolized chemicals only), fat volume and blood flow (for PERC) and cardiac output and alveolar ventilation rate for all chemicals and subpopulations. Intrinsic clearance (=Vmax/Km) is influential for chloroform and TCE, but not for PERC. Although the magnitude of the sensitivity indices varies between the subpopulations for a given parameter, the absolute trend is generally constant. Exceptions are cardiac output (Qc) and alveolar ventilation rates (Qp) in the neonate model for TCE and PERC, as well as for the child in the PERC model only, for which a negative sensitivity index indicates that, contrary to the other subpopulations/chemicals, AUC_pc_ decreases when the parameter value increases.

### 3.2. Variability of Internal Dosimetry for Multi-Route Exposure

Table 3 shows the variability of internal DM in each subpopulation for the multi-route exposure. None of the VI, calculated as the ratio of the 95th percentile value of DM in a given subpopulation to the median in adults, exceeds the default value of 3.16 used in risk assessment [51]. The intra-group variability (as measured by VI_spop_) in subpopulations other than adults is almost always lower than the inter-group variability (as measured by VI). The exceptions were: neonates on the basis of AUC_met_ for PERC, PW based on AUC_met_ for TCE and PERC, and Amet for PERC only. Overall, the greater inter-subpopulation variability based on AUC_pc_ and AUC_met_ is observed when the neonates are accounted for (VI in the range of 2.07–3.12 (AUC_pc_) and 2.30–2.52 (AUC_met_)), and when the children are considered (range: 1.94–2.29) with regard to Amet.

### 3.3. Variability in the LEQ Values

#### 3.3.1. LEQ Based on Absorbed Dose

Table 4 shows the distribution of the LEQ value in each subpopulation according to the absorbed dose of each contaminant under study during the multi-route exposure. In adults, elderly and PW, the inhalation route contributes the most to the absorbed dose of chloroform and TCE, followed by DW ingestion and dermal contact. This is also true in neonates and children for PERC, but not for chloroform and TCE, for which ingestion and inhalation’s ranks are inverted. For the multi-route exposure, mean BW-adjusted LEQ values are greater in neonates regardless of the contaminant considered (0.129–0.134 L/kg BW), as compared to any other subpopulation. In terms of absolute values however, the highest LEQs are computed for PW (4.1–5.6 L). Overall, mean LEQ values for the multi-route scenario are greater for chloroform (4.1 L in a 70 kg adult), followed closely by TCE (4.0 L) and further by PERC (3.6 L). The absolute LEQ values obtained using the 95th percentile value of BW-adjusted LEQ do not increase tremendously, given the relatively low intra-group variability in absorbed dose.

#### 3.3.2. LEQ Based on Internal Dose Metrics

Table 5 shows the distribution of the LEQ values in each subpopulation according to internal DMs of each contaminant under study during the multi-route exposure. Inhalation contributes the most to the AUC_pc_-based LEQ in every subpopulation, followed by dermal exposure except in neonates for TCE and in children for TCE and PERC. Ingestion contributes the most to metabolite-based LEQs for chloroform and TCE, whereas inhalation route contributes the most in the case of PERC. The contribution of dermal exposure to metabolite-based LEQs is always the lowest, except for PERC in adults, PW and elderly. Multi-route LEQ values based on AUC_pc_ are always greater in neonates on a BW-basis (means of 0.190–0.405 L/kg BW) but are greater in terms of absolute values in PW (means of 11.5–28.6 L), followed by adults and the elderly, who share similar values. The same is true on the basis of the other DM, with ranges of absolute mean LEQ values for PW and adults of 3.7–6.6 and 3.0–5.8, respectively, on the basis of Amet and of 4.3–11.9 and 3.5–9.2 based on AUC_met_. The consideration of the 95th percentile value of BW-adjusted LEQs does not significantly change the value observed given a relatively low variability, except in the case of metabolite-based LEQ for PERC (Table 5). Actually, PERC exhibits the greatest value of metabolite-based LEQ values (9.2 and 5.8 in adults on the basis of AUC_met_ and Amet, respectively) as compared to the other contaminants, while LEQ based on AUC_pc_ for chloroform was the highest (mean of 23.5 L in adults, as compared to 8.8 L and 8.7 L for TCE and PERC, respectively).

## 4. Discussion

The goal of the present work was to evaluate the inter-individual variability of internal dosimetry and LEQ values during multi-route exposure to three known DWCs. To do so, the model described previously [21] was used given its capability to use age- and gender- specific equations to define physiological parameters, thus reflecting the inter-individual variability in the critical determinants of toxicokinetics [52,53]. In this regard, the results obtained herein are consistent with the known subpopulation-specific differences in these determinants. Indeed, in neonates, greater-than-adult blood concentrations of parent compound (and TCA resulting from highly metabolized TCE) is likely explained by a greater intake on a BW-basis (Figures 1, 2) [53], whereas children’s greater TCA levels from the low (thus enzyme-limited) metabolism of PERC likely result from the age-related differences in clearance combined with greater-than-adult intake. In addition, differences in TCA blood levels in the elderly and PW as compared to adults are consistent with the respective renal functions in these subpopulations [20,26]. Overall, the results taken together suggest that the LEQs on the basis of absorbed dose or metabolic dose metrics simulated in this study are comparable to the values used in setting the guideline values for these DWCs.

Current results are also consistent with the premise that the variability of internal dosimetry during multi-route exposure is within the range of the variability measures obtained for each route taken separately. Indeed, the VI values reported in Table 3 are generally within the range of the route-specific human kinetic adjustment factors (HKAF) obtained in previous work [21]. The exceptions are VI obtained in children on the basis of Amet of chloroform (2.05 *versus* range of 1.1–1.8) and TCE (1.94 *versus* range of 1.1–1.8), as well as in neonates on the basis of AUC_pc_ of TCE (3.12 *versus* range of 2.2–3.1). Presumably, this is due to the significantly greater water ingestion rate, on a BW basis, in neonates and children as compared to adults, which was not typically accounted for by the HKAF for oral exposure [21]. When the hepatic metabolism overcomes the increased intake, as occurs in children, it results in a greater amount of parent compound being metabolized and a corresponding Amet-based VI. The VI for adults exceeding the HKAF range in the previous work based on AUC_met_ for PERC (2.18 *versus* range of 2.0–2.1) is likely the result of inherent variations from one MC simulation to another. Overall and regardless of the DM considered, the intra-subpopulation variability appeared rather low, as shown by the various CV obtained for LEQ values (Tables 4–5). Exceptions were with the metabolite-based DM of PERC (Table 5). Presumably, this exception is due to the low levels of metabolite generated by the biotransformation of PERC, which makes them more sensitive to any variation in the determinants of metabolism kinetics.

The results of the current study with regard to the LEQ value obtained in an average 70 kg adult based on the absorbed dose corresponds very well with the values obtained in other studies. In particular, the mean LEQ values for multi-route exposure to chloroform (4.1 L) and TCE (4.0 L) are identical to the values obtained by Krishnan and Carrier [18], which were retained by Health Canada [14,15] in its determination of DWG for these contaminants. They are also comparable to the values obtained by Haddad *et al.* [9] for a 30 min shower, *i.e.*, 4.65 and 4.54 L for chloroform and TCE, respectively. The inhalation of vapors during showering contributed to 1.71 and 1.95 L for chloroform and TCE respectively, while corresponding numbers in this study are 1.61 and 1.89 L. Dermal contact during showering with water containing chloroform and TCE was reported to contribute 1.44 and 1.08 LEQ in Haddad *et al.* [9] compared to 1.05 and 0.77 LEQ in the present study.

Based on internal dose, the results obtained also correspond well to the values of Haddad *et al.* [9], as the mean multi-route LEQ values based on AUC_pc_ were 23.5 and 8.8 L for chloroform and TCE in the current study, as compared to 24.0 and 8.5 L. Based on Amet, the corresponding values were 3.2 and 3.0 L as compared to Haddad *et al.*’s 3.69 and 3.57 L [9]. No LEQ values were calculated for PERC by Rao and Brown [11], but the maximum blood concentration at the end of the 30 min exposure to water containing 1 mg/L of PERC via the dermal and inhalation routes only (11–13 μg/L) corresponds roughly to the two-thirds of the maximum venous blood concentration obtained in this study with the adult model (18 μg/L), for corresponding exposure (not shown).

Differences in dose-metrics and chemical-specific LEQ values can be explained based on toxicokinetic mechanisms. Indeed, greater LEQ based on AUC_pc_ than on the absorbed dose can be explained by the fact that during inhalation and dermal exposure, chemicals are not subject to the hepatic first pass effect, as opposed to when entering the body through ingested water. Thus, a greater dose reaches bloodstream and AUC_pc_-based LEQ increases correspondingly (Table 5). This difference is not accounted for when considering only the absorbed dose to establish LEQ. The effect is stronger for chloroform than TCE, as chloroform is more extensively metabolized and presents a slower oral absorption rate (K_o_) such that the hepatic metabolic capacity is not overwhelmed.

The effect of the first-pass metabolism on the LEQ depends upon the dose metric chosen for the assessment. Since a greater amount of parent compound is subject to metabolism during ingestion than when inhaled or absorbed by dermal contact, multi-route LEQ values are likely to be smaller when based on the amount metabolized than on the absorbed dose. This reasoning is however conditional to quick oral absorption. Otherwise, such as for PERC, the LEQ values based on metabolite-based DM depends of the hepatic metabolism that occurs because of the income of parent compound in the blood via the hepatic artery from the systemic circulation rather than from the gut content via the portal vein. As a result, the metabolite-based LEQs are greater than those based solely on the absorbed doses (Table 5), due to higher amounts of parent compound being metabolized during inhalation and dermal exposure.

The toxic moiety on which the LEQ values are based needs to be assessed appropriately, should the results be used for regulatory purposes. Thus, even though high LEQ values were obtained in the present study on the basis of AUC_pc_, the adverse effects underlying the VTR of these chemicals are attributed to the metabolite DM [38,43,47,54], for which LEQ values were lower than the values considered by the regulatory agencies [13–16]. Also, the data, and time span covered by the respective age range, for each subpopulation, have to be considered in relation to the duration for which the guidelines are aimed to provide coverage [55].

Several sources of uncertainties are associated with the present study. First, bathroom exposure duration (30 min) appears to be a major one since it directly impacts the total dose absorbed, but this assumption errs on the side of greater LEQ values and safety due to greater contributions by dermal and inhalation routes. Second, the K_o_ values were extrapolated from animal data for chloroform and PERC and this is a fundamental issue since it strongly influences the internal dose metrics for ingestion and thus the LEQ values. There might also be some uncertainty relating to the use of the same value for partition coefficients (PC) in the PBPK models for all subpopulations despite the fact that tissue composition varies somewhat with age and physiological state [20,52,53]. However, the data from Mahle *et al.* [56] have suggested that PC values do not vary significantly with age. Third, the results of the sensitivity analyses presented also pointed out that several physiological determinants also impact the toxicokinetics and internal dosimetry during multi-route exposure. Focusing to get better estimates of these parameters would translate into corresponding certainty in the results obtained herein, in particular for highly sensitive parameters, for which the impact varied as a function of the chemical and subpopulation considered. For example, a negative sensitivity index for Qp in enzyme-impaired neonates for poorly metabolized PERC is due to the main contribution of pulmonary clearance to overall systemic clearance whereas for highly metabolized chloroform, blood-flow limited hepatic clearance is the main contributor to total systemic clearance. As a result, Qp rather contributes to the intake of chloroform, as shown by a positive sensitivity index, in the same way as it does for each chemical in adults. Fourth and finally, variability in BW-adjusted water intake was not accounted for in the MC simulations, but this variability is likely to be more reflective of differences in personal habits and environmental conditions rather than variability in the physiological and metabolic capacities among people. Future work integrating probabilistic models of human activity pattern, environmental distribution and phamacokinetics might facilitate the simulation of LEQ distributions associated with various multi-route exposure scenarios in subpopulations of interest. However, advanced statistical tools and algorithms for interpreting and dissecting the contributions of the various sources of variability should be in place to facilitate a meaningful interpretation of the MC simulations of LEQ distributions.

## 5. Conclusions

This study has for the first time systematically examined the variability of DM for DWCs during multi-route exposure in various subpopulations. It has also confirmed that the LEQ values determined based on these DM vary significantly. These considerations should be accounted for in future works regarding the determination of drinking water guidelines for contaminants that present a significant potential for multi-route exposure.

## Figures and Tables

**Table 1:** Probabilistic and deterministic descriptors used to define subpopulation-specific parameters in the PBPK models a).

Subpopulation Median age (range)	Adults 41 (18–64) (a)	Neonates 14 d (0–30 d) (a)	Children (1–3) (a)	Elderly 78 (65–90) (a)	Pregnant women 29 (15–44) (a)
Parameter					
PROBABILISTIC VARIABLES (b)					
Body weight (kg, mean ± SD, range):	76 ± 17, 37–152 (c)	4 ± 1, 2–7	13 ± 2, 7–32 (c)	72 ± 16, 33–155 (c)	82 ± 18, 48–166

Body height (cm, mean ± SD, range):	167 ± 10, 144–198 (c)	46 ± 16, 35–80	87 ± 6, 70–106 (c)	164 ± 10, 138–190 (c)	161 ± 7, 132–182 (c)
CYP2E1 concentration (pmol/mg MSP, mean ± SD):	49 ± 2, 11–130 (d)	18 ± 14, 1–56	42 ± 18, 18–74	(e)	(e)
DETERMINISTIC VARIABLES					
Glomerular filtration rate (mL/min_1.73 m^2^)	116.0	40.2	127.0	92.4	181.0
Drinking water ingestion rate (mL/day-kg BW)	19.9	52.4	46.8	21.8	21

REFERENCES	[28,30,33]	[27,32,33]	[27,33]	[28,30,33]	[28,30,31,34]

Notes:

(a) See Valcke and Krishnan [21] for details.

(b) Log-normally distributed.

(c) P^3^M database (see [29]).

(d) Geometric mean ± geometric standard deviation.

(e) Same as for adults. Abbreviations: BW = body weight; MSP = microsomal protein; SD = standard deviation.

**Table 2:** Chemical-specific parameters for PBPK modeling.

Parameters	Contaminants		
	Chloroform(a)	TCE(a)	PERC(a)
Molecular weight (g/mol)	119.38	131.2	165.8
Transfer efficiency in the shower stall	0.534	0.61	0.66
Absorption constants			
Oral (min^−1^/kg^−0.25^)	0.032	0.1667	0.00216
Dermal (cm/min)	0.00267	0.002	0.00207
Urinary excretion constant of TCA (min^−1^/kg^−0.25^)	-	0.0012	0.0012
Partition coefficients			
Blood:air	7.43	9.2	11.58
Liver:air	17	62.56	61.14
Fat:air	280	671.6	1449.8
Highly perfused tissues:air	17	62.56	58.7
Rest of the body:air	12	21.16	70.6
Skin:air	12	20.26	275.2
Kidney:air	11	-	58.7
Water:air	3.66	0.83	0.79
Placenta:blood	2.2	2.7	3.2
Metabolic constants			
Maximal rate (μg/min/kg^0.75^)	211.33	166.67	4
Vmaxc proportionality constant kidney/liver	0.033	-	0.1
Michaelis-Menten (μg/L)	448	1500	7700
Fraction of metabolism in TCA		0.25	0.6
Volume of distribution of TCA	-	0.1 × BW	0.1 × BW

REFERENCES	[9,35–38]	[9,36,37, 39–43]	[11,36,37, 39–41,44–46]

Notes:

(a) See Valcke and Krishnan [21] for details. Abbreviations: PERC = tetrachloroethylene; TCA = trichloroacetic acid; TCE = trichloroethylene.

**Table 3:** PDF for internal dose metrics and resulting variability indices (VI) obtained for multi-route exposure scenario in each subgroup.

Contaminant	Chloroform		Trichloroethylene			Tetrachloroethylene		
Subpopulation	24-h AUC_pc_	24-h Amet	24-h AUC_pc_	24-h AUC_met_	24-h Amet	24-h AUC_pc_	24-h AUC_met_	24-h Amet
Adults								
Median	16.4	22.5	25.4	1104	20.3	50.2	41.3	0.35
95th percentile	21.3	27.4	36.1	1346	25.5	66.8	89.9	0.76
**VI and VIspop**	**1.30**	**1.22**	**1.37**	**1.22**	**1.26**	**1.33**	***2.18***	**2.17**
Neonates								
Median	32.5	31.6	58.4	1726	22.1	97.3	34.4	0.20
95th percentile	47.2	39.9	79.2	2539	32.7	103.8	103.9	0.57
VI_spop_	1.45	1.26	1.36	1.47	1.48	1.07	3.02	1.25
**VI**	**2.89**	**1.78**	***3.12***	**2.30**	**1.61**	**2.07**	**2.52**	**1.63**
Children								
Median	23.6	37.9	39.9	1566	32.4	81.2	50.5	0.46
95th percentile	28.3	46.1	49.2	1866	39.4	92.1	89.9	0.80
VI_spop_	1.20	1.22	1.23	1.19	1.22	1.13	1.78	1.74
**VI**	**1.73**	***2.05***	**1.94**	**1.68**	***1.94***	**1.83**	**2.18**	**2.29**
Elderly								
Median	16.4	23.6	26.2	1222	21.3	51.2	45.8	0.36
95th percentile	21.4	28.0	35.6	1493	27.1	67.5	96.2	0.75
VI_spop_	1.30	1.19	1.36	1.22	1.27	1.32	2.10	2.08
**VI**	**1.31**	**1.28**	**1.40**	**1.35**	**1.33**	**1.34**	**2.33**	**2.14**
Pregnant women								
Median	17.4	26.5	26.8	976	23.4	48.6	32.8	0.34
95th percentile	21.9	32.9	35.4	1181	29.9	60.2	68.3	0.72
VI_spop_	1.26	1.24	1.32	1.21	1.28	1.24	2.08	2.12
**VI**	**1.34**	**1.47**	**1.39**	**1.07**	**1.48**	**1.20**	**1.66**	**2.06**

**Notes**: Underlined values denote subgroup with the highest VI for corresponding internal dose surrogate. Italic denotes VIs that are greater than the range defined by the corresponding HKAFs obtained by Valcke and Krishnan [21] for each exposure routes taken separately.  **Abbreviations**: Amet = amount metabolized for 24 hours and normalized to liver volume (μg/24 h.L of liver); AUC = area under the arterial blood concentration *versus* time curve (μg.24 h/L); met = circulating metabolite; pc = parent compound; spop = subpopulation; VI = variability index as the ratio of the 95th percentile on the median in adult (VI) or in subpopulation (VI_spop_).

**Table 4:** Variability of chemical-specific LEQ in each subpopulation based on the absorbed dose for the multi-route exposure scenario.

LEQ distributions (L/kg BW)	Subpopulation				
**Contaminant** Exposure route	Adults (70 kg)	Neonates (4 kg)	Children (10 kg)	Elderly (70 kg)	Pregnant women (82.5 kg) (b)
**Chloroform**					
Inhalation (mean, CV)	0.023, 21%	0.045, 25%	0.035, 18%	0.023, 21%	0.028, 23%
Dermal (mean, CV)	0.015, 13%	0.039, 21%	0.027, 12%	0.015, 13%	0.013, 12%
Ingestion (mean, CV)	0.020, 22%	0.050, 28%	0.048, 17%	0.022, 21%	0.022, 21%
Multi-route (MR) (mean, CV)	0.058, 19%	0.134, 24%	0.110, 15%	0.061, 18%	0.063, 19%
MR LEQ for BW (mean, 95th perc.)(a)	4.06, 5.48	0.54, 0.78	1.10, 1.37	4.25, 5.64	5.16, 6.91
**Trichloroethylene**					
Inhalation (mean, CV)	0.027, 21%	0.052, 25%	0.040, 18%	0.027, 21.0%	0.032, 24%
Dermal (mean, CV)	0.011, 13%	0.029, 18%	0.020, 12%	0.011, 12%	0.010, 11%
Ingestion (mean, CV)	0.020, 22%	0.053, 25%	0.047, 17%	0.022, 21.0%	0.025, 19%
Multi-route (MR) (mean, CV)	0.058, 20%	0.134, 24%	0.107, 15%	0.060, 19%	0.067, 19%
MR LEQ for BW (mean, 95th perc.)(a)	4.04, 5.49	0.54, 0.79	1.07, 1.36	4.23, 5.75	5.56, 7.00
**Tetrachloroethylene**					
Inhalation (mean, CV)	0.030, 22%	0.058, 23%	0.044, 18%	0.030, 21%	0.036, 23%
Dermal (mean, CV)	0.012, 14%	0.029, 22%	0.021, 12%	0.012, 13%	0,011, 12%
Ingestion (mean, CV)	0.010, 21%	0.042, 23%	0.033, 15%	0.011, 17%	0.010, 19%
Multi-route (MR) (mean, CV)	0.051, 18%	0.129, 22%	0.098, 14%	0.053, 18%	0.057, 19%
MR LEQ for BW (mean, 95th perc.)(a)	3.60, 4.69	0.52, 0.74	0.98, 1.21	3.70, 4.90	4.71, 6.35

Notes:

(a) obtained by multiplying the BW-adjusted LEQ value by the indicated BW.

(b) Pregnant women's BW is based on the mean BW for an adult women as per the P^3^M database to which the mean increase in BW during pregnancy (as per ICRP [31]) was added. Abbreviations: BW = body weight; CV = coefficient of variation; LEQ = litre-equivalent; MR = multi-route; MR LEQ = multi-route litre-equivalent.

**Table 5:** Variability of chemical-specific LEQ in each subpopulation based on internal dose metrics for the multi-route exposure scenario.

	LEQ distributions in each subpopulation (L/kg BW)							
Contaminant Dose	Chloroform		Trichloroethylene			Tetrachloroethylene		
Subpopulation metrics Exposure route	24-h AUC_pc_	24-h Amet	24-h AUC_pc_	24-h AUC_met_	24-h Amet	24-h AUC_pc_	24-h AUC_met_	24-h Amet
**Adults (70 kg)**								
Inhalation (mean, CV)	0.200, 16%	0.016, 22%	0.076, 17%	0.023, 17%	0.017, 22%	0.078, 39%	0.084, 50%	0.048, 100%
Dermal (mean, CV)	0.113, 18%	0.009, 15%	0.029, 20%	0.009, 18%	0.007, 18%	0.028, 21%	0.028, 46%	0.017, 47%
Ingestion (mean, CV)	0.022, 55%	0.020, 24%	0.020, 40%	0.018, 13%	0.020, 25%	0.019, 15%	0.020, 46%	0.020, 50%
Multi-route (MR), (mean, CV)	0.335, 16%	0.045, 19%	0.125, 18%	0.050, 13%	0.043, 21%	0.124, 19%	0.132, 49%	0.082, 47%
MR LEQ for BW (mean, 95th)(a)	23.5, 29.9	3.2, 4.3	8.8, 11.6	3.5, 4.3	3.0, 4.2	8.7, 11.4	9.2, 17.8	5.8, 6.3
**Neonates (4 kg)**								
Inhalation (mean, CV)	0.192, 16%	0.028, 29%	0.100, 14%	0.040, 33%	0.031, 39%	0.094, 2%	0.096, 66%	0.054, 69%
Dermal (mean, CV)	0.138, 17%	0.020, 30%	0.049, 18%	0.020, 35%	0.015, 40%	0.044, 14%	0.043, 67%	0.026, 69%
Ingestion (mean, CV)	0.075, 55%	0.048, 31%	0.063, 32%	0.044, 32%	0.046, 41%	0.052, 10%	0.043, 70%	0.042, 71%
Multi-route (MR), (mean, CV)	0.405, 22%	0.096, 28%	0.212, 19%	0.104, 32%	0.092, 39%	0.190, 4%	0.182, 66%	0.122, 70%
MR LEQ for BW (mean, 95th)(a)	1.6, 2.3	0.4, 0.6	0.9, 1.1	0.4, 0.6	0.4, 0.6	0.8, 0.8	0.7, 1.7	0.5, 1.2
**Children (10 kg)**								
Inhalation (mean, CV)	0.270, 12%	0.022, 10%	0.106, 11%	0.031, 16%	0.025, 18%	0.085, 8%	0.079, 35%	0.046, 37%
Dermal (mean, CV)	0.176, 13%	0.015, 13%	0.049, 14%	0.014, 16%	0.011, 16%	0.037, 14%	0.033, 39%	0.020, 37%
Ingestion (mean, CV)	0.056, 34%	0.047, 17%	0.054, 24%	0.044, 10%	0.045, 19%	0.046, 9%	0.043, 34%	0.043, 35%
Multi-route (MR), (mean, CV)	0.500, 12%	0.084, 16%	0.209, 12%	0.089, 11%	0.081, 16%	0.167, 8%	0.155, 36%	0.110, 36%
MR LEQ for BW (mean, 95th)(a)	5.0, 6.0	0.8, 1.1	2.1, 2.5	0.9, 1.1	0.8, 1.1	1.7, 1.9	1.6, 2.6	1.1, 1.8
**Elderly (70 kg)**								
Inhalation (mean, CV)	0.202, 15%	0.016, 21%	0.077, 17%	0.023, 17%	0.018, 21%	0.077, 16%	0.088, 48%	0.047, 47%
Dermal (mean, CV)	0.114, 18%	0.009, 16%	0.029, 20%	0.009, 18%	0.007, 18%	0.028, 21%	0.028, 48%	0.017, 47%
Ingestion (mean, CV)	0.025, 48%	0.023, 20%	0.023, 35%	0.021, 11%	0.021, 24%	0.021, 13%	0.023, 44%	0.021, 50%
Multi-route (MR), (mean, CV)	0.339, 16%	0.048, 19%	0.129, 18%	0.053, 13%	0.045, 20%	0.126, 16%	0.138, 47%	0.085, 48%
MR LEQ for BW (mean, 95th)(a)	23.7, 30.7	3.4, 4.5	9.0, 11.9	3.7, 4.5	3.2, 4.3	8.8, 11.3	9.7, 18.8	6.0, 11.6
**Pregnant women (82.5 kg)**								
Inhalation (mean, CV)	0.227, 14%	0.018, 24%	0.090, 14%	0.024, 18%	0.019, 25%	0.097, 12%	0.095, 45%	0.053, 49%
Dermal (mean, CV)	0.098, 18%	0.008, 15%	0.026, 20%	0.007, 19%	0.005, 19%	0.027, 20%	0.025, 48%	0.015, 47%
Ingestion (mean, CV)	0.024, 46%	0.021, 23%	0.023, 34%	0.021, 13%	0.021, 24%	0.022, 11%	0.024, 42%	0.023, 48%
Multi-route (MR), (mean, CV)	0.347, 14%	0.046, 20%	0.139, 16%	0.052, 14%	0.045, 22%	0.146, 12%	0.144. 44%	0.091, 46%
MR LEQ for BW (mean, 95th)(a)	28.6, 35.8	3.8, 5.3	11.5, 14.5	4.3, 5.2	3.7, 5.3	12.1, 14.6	11.9, 22.0	6.6, 12.3

Notes:

(a) obtained by multiplying the BW-adjusted LEQ value by the indicated BW. Abbreviations: Amet, amount metabolized during 24 hours normalized to liver volume (μg/24 h.L of liver); AUC, area under the arterial blood concentration *vs* time curve (μg.24 h/L); BW, body weight; CV, coefficient of variation; met, circulating metabolite; pc, parent compound; LEQ, litre-equivalent; MR, multi-route; MR LEQ, multi-route litre-equivalent.
